# Supplementary material for: Multi‐dataset identification of innovative feature genes and molecular mechanisms in keratoconus
Source: J Cell Mol Med. 2024 Sep 19;28(18):e70079. doi: 10.1111/jcmm.70079 (PMC11412914; doi:10.1111/jcmm.70079)
Supplement: Supplementary file 2 — Material S2. [file JCMM-28-e70079-s002.zip › 7.GSEA analysis/2.ATOH7/KEGG_FRUCTOSE_AND_MANNOSE_METABOLISM.html]

Details for gene set KEGG\_FRUCTOSE\_AND\_MANNOSE\_METABOLISM[GSEA]

|  || Dataset | ATOH7.group.cls#h\_versus\_l |
| Phenotype | group.cls#h\_versus\_l |
| Upregulated in class | h |
| GeneSet | KEGG\_FRUCTOSE\_AND\_MANNOSE\_METABOLISM |
| Enrichment Score (ES) | 0.48063555 |
| Normalized Enrichment Score (NES) | 1.3384554 |
| Nominal p-value | 0.17319588 |
| FDR q-value | 0.34068644 |
| FWER p-Value | 0.942 |
Table: GSEA Results Summary

  

Fig 1: Enrichment plot: KEGG\_FRUCTOSE\_AND\_MANNOSE\_METABOLISM      
 Profile of the Running ES Score & Positions of GeneSet Members on the Rank Ordered List

  

| SYMBOL | TITLE | RANK IN GENE LIST | RANK METRIC SCORE | RUNNING ES | CORE ENRICHMENT || 1 | PFKFB1 | na | 91 | 0.780 | 0.1784 | Yes |
| 2 | AKR1B10 | na | 422 | 0.570 | 0.3012 | Yes |
| 3 | ALDOB | na | 1720 | 0.367 | 0.3490 | Yes |
| 4 | FBP2 | na | 2527 | 0.307 | 0.3970 | Yes |
| 5 | AKR1B1 | na | 3374 | 0.261 | 0.4332 | Yes |
| 6 | KHK | na | 4647 | 0.213 | 0.4459 | Yes |
| 7 | HK2 | na | 5782 | 0.177 | 0.4543 | Yes |
| 8 | GMPPB | na | 6913 | 0.145 | 0.4554 | Yes |
| 9 | PFKFB2 | na | 8355 | 0.111 | 0.4396 | Yes |
| 10 | GMPPA | na | 8757 | 0.103 | 0.4520 | Yes |
| 11 | MTMR1 | na | 9072 | 0.098 | 0.4656 | Yes |
| 12 | PMM1 | na | 9721 | 0.086 | 0.4669 | Yes |
| 13 | PFKFB3 | na | 10752 | 0.070 | 0.4534 | Yes |
| 14 | HK1 | na | 11153 | 0.064 | 0.4566 | Yes |
| 15 | PFKM | na | 11206 | 0.063 | 0.4698 | Yes |
| 16 | MTMR2 | na | 11472 | 0.059 | 0.4759 | Yes |
| 17 | PFKFB4 | na | 11758 | 0.056 | 0.4806 | Yes |
| 18 | ALDOA | na | 12691 | 0.044 | 0.4639 | No |
| 19 | HK3 | na | 13130 | 0.039 | 0.4603 | No |
| 20 | TPI1 | na | 14080 | 0.030 | 0.4399 | No |
| 21 | GMDS | na | 15093 | 0.020 | 0.4154 | No |
| 22 | PFKL | na | 15515 | 0.017 | 0.4072 | No |
| 23 | PHPT1 | na | 16235 | 0.011 | 0.3890 | No |
| 24 | MPI | na | 18144 | -0.005 | 0.3352 | No |
| 25 | PFKP | na | 18830 | -0.011 | 0.3181 | No |
| 26 | MTMR6 | na | 21669 | -0.039 | 0.2454 | No |
| 27 | FPGT | na | 22330 | -0.047 | 0.2372 | No |
| 28 | MTMR7 | na | 24417 | -0.076 | 0.1947 | No |
| 29 | PMM2 | na | 25623 | -0.097 | 0.1824 | No |
| 30 | FBP1 | na | 25972 | -0.103 | 0.1963 | No |
| 31 | ALDOC | na | 26100 | -0.106 | 0.2173 | No |
| 32 | SORD | na | 27376 | -0.135 | 0.2119 | No |
Table: GSEA details [plain text format]

  

Fig 2: KEGG\_FRUCTOSE\_AND\_MANNOSE\_METABOLISM      
 Blue-Pink O' Gram in the Space of the Analyzed GeneSet

  

Fig 3: KEGG\_FRUCTOSE\_AND\_MANNOSE\_METABOLISM: Random ES distribution      
 Gene set null distribution of ES for **KEGG\_FRUCTOSE\_AND\_MANNOSE\_METABOLISM**
